# Barriers and enablers to reduced meat intake and perceptions of sustainable diets, among Los Angeles County adults with low incomes: a qualitative interview study

**DOI:** 10.3389/fpubh.2026.1741901

**Published:** 2026-03-26

**Authors:** Katherine Baker, Avaion Ruth, Graciela Corona Rodriguez, Angela W. Zhang, Wändi Bruine de Bruin, Kayla de la Haye

**Affiliations:** 1 Sol Price School of Public Policy, University of Southern California, Los Angeles, CA, United States; 2 Schaeffer Center for Health Policy & Economics, University of Southern California, Los Angeles, CA, United States; 3 Food Systems Institute, Center for Economic and Social Research, Dornsife College of Letters, Arts and Sciences, University of Southern California, Los Angeles, CA, United States; 4 Department of Human Physiology & Nutrition, University of Colorado Colorado Springs, Colorado Springs, CO, United States; 5 Department of Population and Public Health Sciences, Keck School of Medicine, University of Southern California, Los Angeles, CA, United States; 6 Suzanne Dworak-Peck School of Social Work, University of Southern California, Los Angeles, CA, United States; 7 Spatial Sciences Institute, Dornsife College of Letters, Arts and Sciences, University of Southern California, Los Angeles, CA, United States; 8 Department of Psychology, Dornsife College of Letters, Arts and Sciences, University of Southern California, Los Angeles, CA, United States; 9 Department of Community Health Sciences, Fielding School of Public Health, University of California, Los Angeles (UCLA), Los Angeles, CA, United States

**Keywords:** behavior change, climate change, Los Angeles, low income, meat consumption, nutrition, sustainable diets

## Abstract

**Introduction:**

Because of the environmental impacts of meat production, sustainable eating emphasizes eating less meat, and especially less red and processed meat. Here, barriers and enablers to reducing meat intake, and perceptions of sustainable diets, were examined among adults with low income, who often face barriers to food access and choice. The implementation science framework COM-B (Capability, Opportunity, Motivation, Behavior) model was used to contextualize the findings and inform behavior change strategies.

**Methods:**

20 adults in Los Angeles County with low incomes (<300% FPL) participated in semi-structured qualitative interviews. Interviewees were asked about their meat intake behaviors and perceptions, barriers and enablers to reduce meat intake, including red and processed meat, and perceptions of sustainable diets. Interviews were audio-recorded, transcribed, and analyzed using thematic analysis.

**Results:**

Five major themes emerged: (1) Interviewees expressed positive attitudes toward eating less red and processed meat, (2) Self-efficacy, knowledge, and skills related to selecting and preparing alternatives enabled meat reduction; (3) Health-related concerns related to reduced meat intake, and the high cost of meat alternatives, were barriers to reduction; (4) Interest in sustainable eating was high; However, (5) sustainable diets are not the current norm, and interviewees described competing priorities and perceptions that these diets are unattainable. Barriers and enablers to meat reduction spanned different COM-B components, and barriers to sustainable eating within the “opportunity” and “motivation” components emerged through interviews.

**Discussion:**

Interviewees were interested in consuming more sustainable diets, but faced barriers to reducing their meat intake and adopting sustainable diets. For example, interviewees perceived meat reduction as an important way to save money and benefit their health, yet also expressed concerns related to the unattainability of sustainable diets and had health concerns related to meat reduction. This suggests there is a need for clear communication and education, as well as interventions that strengthen enablers while addressing unique barriers faced by urban populations with low income, such as the high cost and accessibility of plant-based foods.

## 1 Introduction

Global food production is a major driver of climate change, and emits an estimated 21–37% of human-produced greenhouse gases (1–3). Additionally, animal foods emit more greenhouse gases and require more water than most other foods, including their plant-based counterparts (2, 4–7). Sustainable eating therefore involves reducing the environmental impact of one’s diet by lowering the consumption of animal foods. Often there is an emphasis on reducing red (including beef, lamb, pork, veal, mutton, goat) and processed (sausages, bacon, ham, etc.) meats (1–3, 8), as these foods contribute to the emission of large amounts of greenhouse gases (1, 5, 9–11), air and water pollution (5, 12), deforestation, and biodiversity loss (1, 9, 13).

Sustainable diets also need to align with human nutrition needs (3), and there is evidence that reduced red and processed meat intake can also benefit human health. Red meat consumption has been associated with adverse health outcomes in some studies, including increased risk of certain cancers (14, 15), type 2 diabetes (16, 17), and cardiovascular diseases (16, 17). Processed meat consumption has an even stronger association with these disease risks (14, 16, 17) as well as all-cause mortality (17). Thus, sustainable and healthy dietary frameworks emphasize the consumption of plant-proteins (e.g., legumes, beans, soy products, and nuts and seeds), combined with whole grains, fruits and vegetables, while lowering the intake of highly processed foods high in sugars, fats, and salt, and low amounts of meat, especially red and processed meats (1, 3, 18, 19). Population adoption of such diets could meaningfully reduce both environmental impacts of the food system (3), and the prevalence of several diet-related chronic diseases including obesity (20), type 2 diabetes, cardiovascular disease (21), and some cancers (22).

While diets that emphasize plant over animal protein are recommended for sustainability and human health (3), Americans consume an estimated 734–1,200 grams of unprocessed red meat (23, 24), and 272–311 grams of processed meat, per week (23, 25). This far exceeds the EAT-Lancet Planetary Health Diet, a framework for health-promoting and sustainable diets, recommendations for up to 100 grams of red meat, and 0 grams of processed meat, per week (3). Despite current meat intake exceeding recommendations, many Americans report willingness to reduce (26–28). In addition to this willingness, representative survey data suggest that over half of Americans are aware of the global warming impact of some foods, including beef (57%), and pork (55%) (29).

Taken together, existing literature suggests some awareness of environmental impacts of (29), and openness to reduce meat (26–28) among United States (US) adults. However, consumers who want to eat less meat may still face a range of multi-level barriers to meat reduction, including taste preferences, a lack of skills, time, or interest in cooking, low perceived ability to control relevant dietary behaviors, and a lack of support in their built and food environments (30). Even among those who have motivation to reduce, cognitive dissonance may mediate motivations, barriers, and stages of behavior change (31). Strategies to strengthen motivation, and address barriers to meat reduction are needed (31).

One important gap in the existing literature exploring people’s beliefs and behaviors related to meat reduction, including awareness of sustainable diets, motivations to reduce meat intake, and facilitators and barriers to doing so, is a lack of research on the specific needs of priority populations. Segments of the population, including Americans with low incomes, face the greatest risk of diet-related disease, and the most barriers to dietary behavior change (32). Americans with lower income, for example, are more likely to report plant-based foods cost too much (27), and they experience higher rates of food insecurity (33) and barriers to accessing affordable healthy food in their neighborhoods (32), which make dietary change difficult. They may also face unique barriers related to having the time and resources for acquiring plant-based foods, and the social and cultural norms that would support these dietary shifts. A greater understanding of the perspectives, barriers, and enablers to meat reduction among low-income Americans is necessary to develop programs and policies that can enable shifts toward sustainable eating for all, especially those segments of the community who face the greatest burdens of poor diets, and impacts of environment degradation (26, 34).

The present study examines experiences of those with low income in Los Angeles (L. A.) County. L. A. County is a large and diverse urban area with over 9.7 million residents (35), many of whom experience financial struggles that impact their food access. Currently 13.7% of Los Angeles County residents (1.3 million people) live in poverty (36), exceeding the 10.6% national poverty rate (37). In 2024, 25% of households in L. A. County, and 41% of households with low income experienced food insecurity (38), compared to 13.7% of households nationally (39). Additionally, L. A. County residents with low income continue to experience higher rates of diet-related chronic diseases, and only 10.8% of low-income adults consume the recommended 5 or more servings of fruit or vegetables a day (40). To understand the experiences and beliefs related to sustainable eating and reduced meat intake among this priority population, the present study explores the following topics with adults with low income residing in L. A. County:

1. Current meat-eating patterns, and perceptions of meat reduction;
2. Barriers to reducing meat, red and processed-meat;
3. Enablers to reducing meat, red and processed-meat; and
4. Interest in, and perceptions of, sustainable eating generally, which were described as “ways to make food choices that are better for the environment.”

In the context of the current study, given that red and processed meat have such a high environmental impact (1, 3, 5, 9, 12, 41–43), the study aimed not only to understand the above related to meat generally, but also red and processed meat specifically.

### 1.1 Theoretical framework: capability, opportunity, motivation, behavior (COM-B) model

The capability, opportunity, motivation and behavior (COM-B) model identifies domains that are established predictors of behavior change (44). COM-B, originally proposed by Michie et al. (2011), links three segments of “essential conditions” of a behavior system: “capability” (or the psychological or physical ability to engage in a behavior), “opportunity” (factors outside of an individual that impact the ability to partake in a behavior) and “motivation” (mental processes that direct and drive the behavior) (45). COM-B articulates these behavior system phenomena as an interacting and dynamic system, where these three essential conditions can influence behavior directly, or through complex interaction, and this effects on behavior may in turn influence future conditions (45). For example, positive “opportunity” could promote the adoption of a behavior, as could increased “capability.” And combined, positive “opportunity” and increased “capability” could heighten “motivation,” which in turn can further support behavior change (45), and ultimately the conditions of the behavior system.

The COM-B model has been used as an overarching framework to understand barriers and enablers to reduce meat, and increase plant-based eating (46); such efforts have identified that much existing literature on meat reduction focuses on the “motivation” component, and that additional research into “capability” and “opportunity” variables is needed (46). In the present study, this framework is applied post-analysis to contextualize barriers and enablers to meat reduction behaviors, and to understand challenges related to sustainable eating.

## 2 Methods

### 2.1 Design

This study employed a qualitative and inductive approach to characterize interviewees’ current meat eating habits and beliefs about reducing meat intake and sustainable eating. Semi-structured interviews were conducted with twenty adults in L. A. County who had low incomes. The protocol was reviewed and approved by the University of Southern California’s Institutional Review Board [under review 45 CFR 46.110(b)(1)(ii)].

### 2.2 Participants

Interviewees were recruited from the representative L. A. County sub-sample of the University of Southern California’s Understanding America Study (UAS). The UAS is a nationally representative online panel that recruits adults aged 18 years and older through random address-based sampling (47), and participants are provided with a tablet and/or internet access if needed to complete surveys (47). Study participants were eligible for participation in our interviews if they lived in L. A. County, and had household incomes below 300% of the federal poverty level (FPL) based on 2023 State Income Limits from the State of California Business for Los Angeles County (48). Interviews took place in March–April 2024, before 2024 State Income Limits were released (49). We used a stratified sampling strategy to capture diverse experiences, aiming to recruit approximately 50% women, approximately 50% with children in the household, approximately 20% self-identified as Black or African American, and 50% as Hispanic or Latino. All interviewees endorsed eating some type of meat and/or seafood/fish. Demographics of interviewees included in the analysis can be found in Table 1.

**Table 1:** Demographic characteristics of interviewees.

Demographic characteristic	*N*
Race/ethnicity	
Non-Hispanic white	8
Non-Hispanic Black	3
Non-Hispanic Asian	3
Hispanic or Latino	5
All other (mixed race, American Indian, Alaska Native, Hawaiian/Pacific Islander)	1
Gender	
Female	10
Male	10
Income status	
Low income (<300% FPL)^*^	20
Highest level of education	
Bachelor’s degree or higher	8
Less than a bachelor’s degree	12
Employment status	
Currently employed	8
All other (unemployed, retired, disabled, mixed, other)	12
Age categories	
18–64	15
65 and over	5

*All participants met criteria to be experiencing a low income based their household income and size, and cut points defined by the Los Angeles County 2023 State Income Limits from the State of California Business, Consumer Services and Housing Agency Department of Housing and Community Development.

### 2.3 Procedure

A semi-structured qualitative interview guide was developed in English to explore interviewees’ views and experiences on the following topics: (1) food preferences and eating habits, (2) meat consumption, (3) perceptions of and willingness to reduce meat intake, and (4) perceptions of sustainable eating, which were described as “ways to make food choices that are better for the environment.” Interview questions about meat asked about both meat generally as well as red and processed meat specifically. The interview guide was reviewed by members of the research team and piloted with non-interviewee volunteers. Revisions were made to improve the flow and clarity of the interview guide. Fourteen open-ended questions were included in the final interview guide, with probes used to further understand the interviewee experience. Sample questions and probes can be found in Table 2. The complete interview guide can be found in Supplementary File 1. The Spanish translation, which was created by UAS staff and reviewed by a Spanish-speaking member of our research team, can be found in Supplementary File 2.

**Table 2:** Sample questions and probes.

Food preferences and eating habits How hard or easy is it for you to eat the types of foods you would like to eat? When you think of your ideal way of eating, what does that look like for you? What makes it easier for you to eat the foods you would like to eat? What makes it harder for you to eat the foods you would like to eat?
Meat consumption How do you feel about the amount of meat you eat? Are you generally happy about the amount of meat you eat? If you could change the amount or type of meat you eat, what kinds of changes would you make? How do feel about the amount of red meats like beef, lamb and pork, that you eat? What has the biggest influence on the amount of meat you eat?
Perceptions of and willingness to reduce meat intake If you were going to reduce the amount of meat you eat, do you think you would be able to do it? Why or why not? What kinds of meat would you reduce? Why? Do you have the information you’d need on how to select or prepare meals that have less meat?
Perceptions of sustainable eating How important do you think it is to think about how the foods you eat impact the environment? When you think about the foods that you typically eat, how environmentally sustainable do you think the way you eat is?

The full interview guide can be found in Supplementary File 1 in English, and Supplementary File 2 in Spanish.

Twenty-one eligible participants were invited into the study by UAS staff. They were told that the study’s purpose was to understand eating habits, and their thoughts about the impact of food on the environment. One participant did not complete the interview. A total of 20 participants completed the interview; 20 participants was selected as a target sample size given that saturation is typically reached in qualitative interviews between the 9–17 interview (50). Interviewees provided consent online before scheduling interviews. They were offered the option to complete the interview in English or Spanish, and interviews were conducted over the phone by trained qualitative researchers and audio-recorded. Data saturation, or the point at which data collected reflects previously collected data (51), was achieved after seventeen interviews; interviews continued to 20 to approximately meet the stratified sampling strategy to collect a diverse set of experiences. Interview recordings were professionaly transcribed for the analysis. The lead researcher (KB) listened to all recorded interviews while recording the transcripts to check for accuracy and made minor corrections to the transcripts as necessary.

### 2.4 Data analysis

Descriptive statistics for demographic characteristics of interviewees were calculated using STATA 17.0. Qualitative data analysis was completed by two researchers. At the time of analysis, KB was a postdoctoral researcher trained in mixed-methods with background in public health, nutrition, environmental health science, and psychology, while AR was a Master of Public Health Student with a background in nutritional epidemiology, food justice, and health disparities. Principles of thematic analysis were used with a realist or essentialist approach, which aims to report experiences and realities of participants (52).

The six steps of thematic analysis used in our study, and outlined by (52), are listed next (52):

1. Data familiarization: KB and AR read each transcript multiple times.
2. Generating initial codes: KB and AR independently used principles of open coding, of the analytic process used to identify concepts, their properties, and dimensions in data (53) in NVivo 17.1 to generate initial codes. Transcripts were coded line-by-line in NVivo; blind coding was not employed. Based on open coding and discussions, a codebook was developed using primarily inductive coding, derived from the open coding process. To test for inter-rater reliability, the coders set an *a priori* goal of reaching 80% coding agreement on 10% of the transcripts. This was achieved after coding three randomly selected transcripts. The researchers met to discuss coding and resolved discrepancies and improved clarity of codes through discussion. The codebook was updated based on discussions throughout the reliability testing process to improve clarity, reduce redundancy, and improve comprehensiveness of codes and definitions. The updated codebooks were used to re-code previously coded transcripts. The final codebook was agreed upon by the two coders, and was then applied to the remaining transcripts, divided between the two coders.
3. Searching for themes: Codes were sorted into potential themes and sub-themes using tables, revisited and refined.
4. Reviewing themes: Themes were reviewed and refined by the coders.
5. Defining and naming themes: KB drafted themes, and AR reviewed and provided feedback on the themes until consensus was met. Additional authors (WBB, KH, AZ) contributed to their refinement.
6. Producing the report: Finalized themes are presented in this manuscript; each finalized theme has a minimum of two supporting interviewee quotes, which are included in this manuscript.

The COM-B framework was used to interpret barriers and enablers to reduce meat intake and the consumption of sustainable diets. Barriers and enablers to sustainable eating were not asked about explicitly, but these topics emerged through the interviews. Application of the COM-B framework was flexible so that topics and themes that may fall outside of this theory and evidence-informed model were not excluded (54).

## 3 Results

### 3.1 Qualitative themes

**Figure 1:**
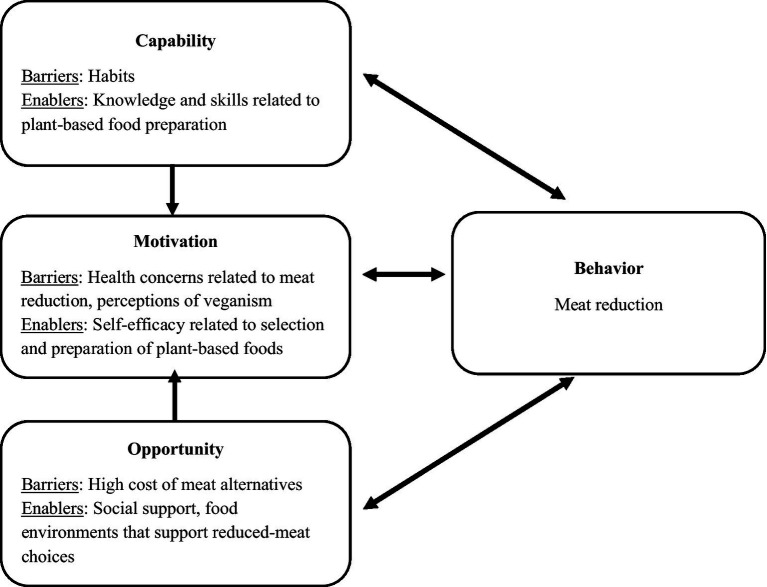
The capability, opportunity, motivation, behavior (COM-B) model, originally proposed by Michie et al. (45), adapted to detail barriers and enablers to meat reduction described among interviewees living with low income in Los Angeles County.

Themes are summarized in Table 3 and detailed below. Key findings related to barriers and enablers of meat reduction are outlined in Figure 1. Potentially identifying details were redacted to protect anonymity.

**Table 3:** Key themes, based on 20 qualitative interviews with adults with low income living in L. A. County.

Key themes	
1	Interviewees had positive feelings toward eating less red and processed meat.
2	Knowledge, skills, and self-efficacy related to selecting and preparing alternatives enabled meat reduction.
3	Barriers to meat reduction included health-related concerns, and the high costs of alternatives.
4	Interest in sustainable eating was high.
5	Sustainable eating was not the current norm or a priority.

#### 3.1.1 Theme 1: interviewees had positive feelings toward eating less red and processed meat

Generally, interviewees expressed positive feelings toward reducing their meat intake. For example, one interview stated: “I feel good about eating less meat,” (*interview 16*). Another said regarding meat reduction: “I’ve cut down a lot, so I have a lot more energy…. I do feel better about it,” (*interview 5*). Preventing or managing health conditions was a primary reason for reduction. One interviewee stated the following regarding what motivated their meat reduction: “The reports of…medical studies that red meat can contribute to…intestinal cancer…and cholesterol,” (*interview 1*). Others reduced meat intake to manage specific health conditions they had currently or in the past. For example, one interviewee said: “Sausages is processed, so [to manage a health condition] the dietitian does not want us to eat processed meats.” (*interview 13*).

Additional reasons for reducing meat intake included feeling physically better when consuming less or no meat, with participants describing positive experiences with less meat consumption and negative experiences with more. For example, one interviewee stated: “whenever I eat meat I feel sluggish and heavy…it does not give me the – the same energy that the vegetables and the fruits do” (*interview 20*). Another interviewee described the impact of meat on digestion: “I know if I eat too much meat, I feel it…in my stomach, in my intestines…I’ll feel bloated,” (*interview 14*). Animal-welfare and environmental reasons were also mentioned: “I think there are health concerns, there are environmental concerns, and there are humanitarian concerns…animal rights concerns,” (*interview 19*). Another interviewee stated: “…as far as beef goes, they say cows are killing the ozone layer…I’m just not one of those people that benefit from slaughtering cows and beef and pork and all that stuff, so hopefully I’m helping to do my part,” (*interview 9*). Interviewees also described how eating less meat helped them save money: “…. I’ve been trying to budget around and, so [eating less red meat has] saved me a little bit of money,” (*interview 3*). Another interviewee stated, regarding meat affordability in the context of other expenses: “inflation is killing us out here in California…we cannot afford it…We’ve [have to] either afford meat or gas. And people would take the gas before the meat,” (*interview 8*).

#### 3.1.2 Theme 2: knowledge, skills, and self-efficacy related to selecting and preparing alternatives enabled meat reduction

Social support and access to alternatives further enabled meat reduction. Overall, enablers to meat reduction fell within “capability,” “motivation,” and “opportunity” components of the COM-B model. Within the “capability” component, interviewees described how having knowledge and skills related to selecting and preparing alternative non-meat/plant-based foods and meals enabled them to consume less meat. For example, one interviewee shared: “it’s easier when I come up with different ideas…I cook it. I find little recipes…all of a sudden that’s what makes it easier not to have meat, is that I can come up with stuff like that [meatless options]” (*interview 6*). With regards to what enabled meat reduction, another interviewee said: “Learning other alternatives for protein, learning how to combine other carbs, legumes, other types of beans, tofu, that are just as comparable to the amount of protein we would get in meat,” (*interview 12*). Having knowledge and skills was complemented by perceptions of self-efficacy to engage in these behaviors. Self-efficacy, first conceptualized by Albert Bandura (55), encompasses one’s perception of their capability to perform a particular behavior in a given setting, or to attain desired results (56). Many health behavior theories view self-efficacy as a primary determinant of behavioral change, and it is a construct of “motivation” component of COM-B (44). Among interviewees who had already reduced their meat intake, they described how they felt capable of doing this. For example, an interviewee stated: “…I understand how to substitute…other things into my diet to get the right amount of protein…I know how to cook very well to not use meats or anything like that,” (*interview 11*). Stated another: “I know my way around the kitchen…I can make tasty meals without having meat in them,” (*interview 20*).

In addition to enablers within the “capability” and “motivation” components, enablers were also described within the “opportunity” component. For example, social influence of family and friends who reduced or eliminated meat impacted interviewee eating habits. For example, an interviewee stated, regarding meat reduction: “probably the biggest thing is that my [significant other] does not eat meat at all,” (*interview 19*). Another interviewee stated: “I have friends and stuff like that who are very health conscious and they are trying to keep me health conscious. And they try to limit how much meat I eat…so I think they have a really positive impact on how much meat I consume,” (*interview 17*). Food environments that supported reduced meat choices were also described as enabling by interviewees, within the “opportunity” component of COM-B. One interviewee said: “being able to go to places, like [a restaurant] that I know that has very healthy plant-based diet or plant-based dishes and, and also going to places like [grocery store], where they have a lot of already made things that do not have any meat that makes it easier.” (*interview 14*). Stated another: “Now…there are so many other options available that are just as tasty, and they are better for you and the environment,” (*interview 20*).

#### 3.1.3 Theme 3: barriers to meat reduction included health-related concerns, and the high costs of alternatives

When asked about their views on reducing meat intake, interviewees noted the health benefits of eating meat, and shared concerns that reducing meat intake could have negative health consequences; a behavioral barrier within the “motivation” component of COM-B. For example, one interviewee said: “I think muscle is very important…so I think it’s a little bit necessary to eat certain meat amount,” (*interview 10*). Another stated: “…you are supposed to have a certain amount of meat,” (*interview 4*). These beliefs about the negative consequences of reducing meat intake were also reinforced by social influences, within the “opportunity” component of COM-B (44). For example, an interviewee described their healthcare provider’s influence on this belief: “I talked to my doctor, I even said I’m going to start going more vegetarian…and [they] said…I need to eat some meat,” (*interview 17*).

The high cost of foods that could be alternatives to meat was also noted as a barrier to reducing meat intake, within the “opportunity” component of COM-B. For example, one interviewee described the high cost of meat alternatives: “…substitutes are very expensive compared to actually just buying meat…” (*interview 17*). Another described the challenge of finding and affording other foods, stating: “[Money] impacts [the ability to eat less meat] a lot, because the vegetables are expensive, too…trying to replace it with other things that can be kind of expensive,” (*interview 4*).

Finally, habits were identified as a reason for continued meat consumption. Habits fall within the “capability” component (44, 101), and refer to ingrained, automated, or impulse-driven processes and routines driven by external or internal cues, which prompt behavior. For example, one interviewee stated: “I always grew up eating meat…I’ve always eaten meat all my life,” (*interview 18*); and another said: “it’s something we grew up with…there are some good memories associated with it” (*interview 12*). These examples indicate behavioral repetition, in combination with positive affect of the behaviors, can result in an automized behavior (57), in this case, eating meat.

##### 3.1.3.1 Sub-theme 3a: negative perceptions of veganism arose in the context of meat reduction

Negative perceptions of veganism were described, despite veganism not being brought up by the interviewer or interview guide. Veganism was generally perceived as unhealthy or unappealing, indicating that such attitudes serve as a barrier within the “motivation” component of COM-B. For example, regarding health impacts of a vegan diet an interviewee stated: “You know, it’s so funny, [someone I know] went vegan, and – and now I think [they are] not doing too well. I just feel like, you know, I’m healthier. I hardly ever get sick,” (*interview 6*). Another stated: “I have friends who are vegans…they get an injury [and] they never heal,” (*interview 17*). Other interviewees expressed desires to avoid vegan diets. For example, stated on interviewee: “I mean this strongly, I do not believe in a vegan or a complete vegan or vegetarian diet,” (*interview 1*), and another asked, regarding options to reduce meat and maintain health: “What substitutes to use in place of the proteins and minerals and stuff that I do need from the meat…what good alternatives would be out there besides just going full vegan?” (*interview 5*).

#### 3.1.4 Theme 4: interest in sustainable eating was high

When asked about sustainable eating as a general topic, not specific to meat reduction, interviewees expressed interest. There was a desire to learn more about sustainable diets and how to engage in them. An interviewee stated: “Sustainable eating, gosh, what is that? [That’s] something that I’m intrigued about… I’m very interested in learning more about that,” (*interview 9*). Additionally, one interviewee expressed: “I would be more interested in seeing how sustainable eating can be implemented in a city, where I live,” (*interview 11*). Interviewees expressed some familiarity with sustainable eating, and reported having heard about it from social and environmental contexts, including family, friends, local establishments, media, and internet sources. One interviewee stated: “…it’s part of my upbringing and knowledge of, of what food is and what food could be,” (*interview 11*). Indicating familiarity with the topic, another stated: “…just recently, that’s when I started hearing it [it’s] all kind of new to me…,” and described hearing about it on the news (*interview 6*).

#### 3.1.5 Theme 5: sustainable eating was not the norm or a priority

While interest in sustainable eating was high, interviewees expressed that the sustainability of their diets could be improved. For example, an interviewee described the sustainability of their diet as “Poor…because what we eat, we tend to eat meat or different things, and we are not cautious….” (*interview 3*). Stated another, regarding the sustainability of their eating: “Probably not good…it’s processed and then it has packaging plastic…all this excess cardboard…so probably not good for the environment,” (*interview 4*).

Competing priorities, such as health, price, or family preferences, were described as barriers to sustainable eating, which align with the “opportunity” component of COM-B (44). An interviewee stated: “I would say, it’s not important because I cannot take on the environment. I cannot. I do not wanna take on the pressure of saving the environment,” (*interview 9*). Another interviewee, who ranked the sustainability of their diet as a three on a scale of one to five stated “[it’s] because of the price. It mandates what I eat, the cost,” (*interview 13*). These perceptions furthermore indicate a lack of self-efficacy related to sustainable diet consumption, related to the COM-B “motivation” component (44).

Interviewees indicated that they did not perceive sustainable eating to be the norm or something that others prioritized, reflecting social influence as an additional barrier within the “opportunity” component (44). For example, an interviewee stated: “I do not think it’s realistic right now for – well, I do not think it’s realistic for Americans…I think that Americans are very used to, highly processed foods. We’re already stuck in a certain lifestyle… [It] would be very hard,” (*interview 2*). One interviewee said: “I think that we are not necessarily there, yet, as a society or a community. So people do not really care…” (*interview 11*). Stated another: “In practice, it’s much more difficult because people do not want to go through the hassle or do not have time to go through the hassle to me more sustainable,” (*interview 20*).

## 4 Discussion

### 4.1 Perceptions of meat reduction

Interviewees reported positive feelings toward reducing their meat intake, especially red and processed meat. Nationally representative survey data suggests many Americans are also reducing: in 2025, 69 and 64% of US adults reduced red meat and processed meat intake in the year prior, respectively (58). Interviewees cited several motivating factors for reducing meat consumption. For example, among interviewees, managing or preventing health conditions (e.g., heart disease, cancer) was one motivation for reducing meat intake, aligning with evidence that health is frequently a key motivator for reducing meat (28, 30, 59–63) and red meat intake (58, 64, 65) in research not focused on persons with low income. Additionally, we uncovered motivations related to meat reduction as a means to avoid digestive difficulties, fatigue, or low energy, which have not been extensively documented in literature among populations with low income or otherwise. Furthermore, interviewees identified the importance of saving money by forgoing expensive meat, which adds to evidence suggesting that the high cost of meats is a motivation for reduction (58, 66–68). Finally, moral considerations, including those related to animal welfare or the environment, have both been cited as strong motivators for some groups (e.g., animal welfare may be important for vegetarians and vegans); however, these motivations are often not as important as others, such as health (69), suggesting that their influence on meat reduction behaviors varies.

### 4.2 Barriers and enablers to reduction within the COM-B model

Our findings suggest meat reduction barriers and enablers exist within the “capability,” “opportunity,” and “motivation” components of the COM-B model (Figure 1), aligning with evidence not focused on low income populations (46). Enabling “capability” factors included knowledge and skills of how to select and/or prepare reduced meat or plant-based meals. These enablers have been found to support meat reduction in studies not focused on low income populations (46, 62, 70, 71). Self-efficacy, or feeling capable of preparing reduced meat meals and meat alternatives was also an important “motivation” enabler (70). Our findings add to others suggesting self-efficacy plays a role in intention and action related to meat reduction (46).

Self-efficacy has also been shown to serve as a mediator between perceived food environments and healthy eating among adults with low income in L. A. County, indicating that when people live in food environments that are supportive of healthy eating, self-efficacy increases healthy food intake enabled by the belief that one is able to eat healthy (72, 73). Results of the current study suggest it may also play a role in enabling meat reduction. Additionally, social influence and support were salient “opportunity” enablers among interviewees, building on existing evidence that social ties with those who engage in meat reduction can serve as behavior models or provide social support (46, 74). Interviewees additionally described physical access to meat alternatives as enabling, also within the “opportunity” component. Evidence has shown that a lack of access to plant-based alternatives may still serve as a barrier in adopting more plant-based diets (75); and that increasing the availability and affordability of meat alternatives may provide opportunities to support plant-based eating (30); our findings suggest this may extend to those with low income.

In addition to enablers, interviewees described barriers to reducing meat intake across COM-B components. First, interviewee perceptions about negative health consequences of consuming too little meat exist within the “motivations” component. This finding adds to evidence that perceptions of potential negative health consequences of a reduced or meat-free diet (30, 76), and beliefs about the health benefits of meat (77), are barriers to reduction. This belief was held simultaneously along the belief that meat reduction is beneficial to health, including among those who reported reduction, adding to literature that health can be a motivator for and against meat consumption (78). Some interviewees stated that this belief was reinforced by healthcare providers, despite expert consensus that meat is not essential for health (79). Furthermore, while the high cost of meat alternatives was cited as an additional “opportunity” barrier, complementing existing evidence that those with lower income purchase less plant-based meat alternatives (80), interviewees also described some meats as too expensive. Meating eating was also described as a long-established habit, a “capability” barrier, which has been shown to strongly predict meat consumption (58, 67, 70, 81–83) and is a cited barrier to reduction elsewhere (8, 39, 73). Finally, negative perceptions of veganism served as a “motivation” barrier. Negative perceptions of veganism are common (84) and may be a deterrent to adopting reduced-meat or plant-based diets more generally (65, 85). Strikingly, while veganism was viewed negatively, perceptions of meat reduction were positive, indicating that negative attitudes toward veganism exist concurrently with positive perceptions of meat reduction.

### 4.3 Sustainable eating

We found overall high interest in sustainable eating among interviewees. Interviewees furthermore expressed some familiarity with the topic of sustainable eating, contradicting existing literature that has repeatedly concluded that most consumers have an overall low awareness of the environmental impacts of food (74, 86). Yet, a gap between interest and action emerged. Competing priorities, including personal health, and family or child preferences competed with sustainability concerns; similar factors have been previously observed to make sustainable food choices difficult (26, 81). The cost of foods was another important competing priority, and an “opportunity” barrier. Price is a key driver of food choice (82), and is often perceived as more important than sustainability (30), including among those with low socioeconomic status (81). While analyses have suggested sustainable diets are less expensive than the counterfactual in high-income countries (8, 83, 87), those with low income may experience limited food choice, and find sustainable diets to be too expensive (88). Our results further suggest that a lack of “self-efficacy” regarding perceived capability to consume a sustainable diet may serve as a “motivation” barrier. Attitudes that sustainable diets are unattainable or unrealistic among interviewees echo findings that US adults perceive sustainable diets as attainable only to the affluent (89). Importantly, these perceptions reveal an additional paradox: while some perceive meat reduction, a key component of sustainable diets, as an important way to save money, sustainable diets are commonly seen as out of reach, including financially. Such social norms, or “implicit codes of conduct that provide a guide to appropriate action” (90) related to low sustainable eating engagement, may influence subsequent behavior (91), and serve as wider barrier to sustainable diet adoption.

### 4.4 Future directions

Findings from this study highlight both the interest among low-income adults in reducing meat consumption and adopting more sustainable diets. Multi-level opportunities to support these changes by addressing key barriers and strengthening enablers relevant to this population are warranted. Additional research is needed to explore these predictors of dietary behavior change among a larger sample of adults with low incomes who live in diverse social and geographic settings, and to design and evaluate intervention strategies tailored for these populations.

At the individual level, results suggests that knowledge and belief (“capability”) as well as self-efficacy (“motivation”), can enable meat reduction. Other observational and intervention research suggests that knowledge, skills-based, and self-efficacy efforts can enable diet change including related to meat reduction (30, 46, 84) and healthy eating among those with low income (72, 73). We therefore we suggest future interventions explore the value of education to build knowledge, and the use of repeated skill-building opportunities (e.g., cooking classes) to build self-efficacy. While evidence-based approaches to sustainable eating education for those with low income have been recommended based on work in Italy (85), we suggest additional inquiry in assessing the efficacy of such methods among US-based urban residents. Importantly, our findings indicate that interest in meat reduction and sustainable diets were high, reflecting positive attitudes and “motivation” toward such dietary changes, which may be an important leverage point toward behavior change. Yet, many still view sustainable diets as inaccessible, despite already reducing meat intake, a key component of sustainable diets (3). Therefore, education that clearly links the role of meat reduction in sustainable eating is needed. Furthermore, given concerns related to veganism in the current study, meat reduction and sustainable diet efforts may benefit from careful framing, including labeling focused on environmental and health benefits, rather use of the word “vegan” (92).

Furthermore, at the interpersonal level, our results suggest strengthening “opportunity” enablers to build on social support from family, friends, and healthcare providers, in efforts to reduce meat and shift toward sustainable diets. Building upon research that suggests social influence is supportive of animal product reduction (93), and that healthcare providers can also support plant-based and sustainable diet shifts (75, 94), evaluation of these social opportunities among urban adults with low-income may add to current understanding.

At the environmental level, the high cost of meat alternatives and the inaccessibility of sustainable diets need be addressed. Increasing affordability of plant-based foods may be beneficial, not only for Americans generally, half of whom report they would eat more plant-based foods if they cost less, but particularly for those with low income, who are less likely to be able to afford them (27, 34). Policy changes, such as subsidies, have shown promise in reducing cost (95). To further address financial and access barriers for those with low income, the use of financial incentives for sustainable foods such as fruits, vegetables, and legumes, has been shown to be successful in increasing access among persons with low income in France (96), and similar approaches described in the interview guide were appealing to interviewees. Given the importance of competing priorities as a barrier to sustainable eating engagement, policies that make sustainable eating patterns the default are recommended (97). For example, “green defaults,” the practice of providing plant-based ingredients or meals as the default option, have been shown to be effective as a low-cost intervention strategy in encouraging shifts toward reduced meat and sustainable food consumption without increasing cognitive burden (98, 99). However, there is opportunity to explore their potential as a tool for increasing accessibility of sustainable diets among those with low income.

### 4.5 Strengths and limitations

The small sample size, and the location of L. A. County, a large, urban county located in California, US, significantly limit the generalizability of these findings. Furthermore, all interviewees were experiencing low income and living in a high-income country, limiting the potential generalizability of these findings to other similar populations. As a qualitative inquiry, interviewees may be subject to social desirability bias, particularly given the sensitive nature of these findings (100). Despite these limitations, this study provides in-depth insights into interest in and perceptions of sustainable eating patterns, as well as barriers and enablers to reduced meat diets, among a diverse sample of L. A. County adults. Additionally, this study contextualized findings within the COM-B framework, and identified barriers and enablers to meat reduction within all COM-B components, adding to literature on meat reduction and plant-based dietary shifts, which has historically focused on “motivation” components, and less so on “opportunity” and “capability” (46). Furthermore, this study uniquely focused on adults with low income, adding to needed research focused on sustainable diets among vulnerable groups (34).

## 5 Conclusion

Interviewees, all of whom were adults living in L. A. County with low income, had positive attitudes toward reducing their intake of red and processed meat. These positive beliefs were linked to the perceived health benefits of meat reduction, such as improved digestion and energy, the opportunity to save money, or out of concerns for animals or the environment. Enablers to meat reduction included knowledge, skills, and self-efficacy related to selecting and preparing reduced meat or plant-based meals, as well as positive social influence, and access to meat alternatives within one’s food environment. Interviewees also described many barriers to reducing their meat intake, including concerns about the health consequences of insufficient meat intake, and the high cost of meat alternatives. Overall, interviewees were interested in sustainable eating beyond the focal point of meat reduction, but did not report their own diets as sustainable, due to competing priorities, including cost, and in part due to perceptions that sustainable eating was unrealistic for themselves and others. Considering interviewees reported numerous barriers to sustainable diets within the “opportunity” and “motivation” components of the COM-B model, future research and interventions should explore which multi-level individual, social, and structural barriers to sustainable dietary behaviors need to be addressed in populations with low incomes to make behavior change feasible and sustainable.

## Data Availability

The datasets presented in this article are not readily available to protect participant anonymity. Requests to access the datasets should be directed to kbaker11@uccs.edu.
