# Supplementary material for: Barriers and enablers to reduced meat intake and perceptions of sustainable diets, among Los Angeles County adults with low incomes: a qualitative interview study
Source: Front Public Health. 2026 Mar 26;14:1741901. doi: 10.3389/fpubh.2026.1741901 (PMC13064549; doi:10.3389/fpubh.2026.1741901)
Supplement: Supplementary file 1 [file Supplementary_file_1.pdf]

Introductory remarks:

Thank you for taking the time to participate in this interview. My name is [*researcher name*] and I will be doing this interview today. This study is part of a project that hopes to understand eating habits.

Logistical information:

This interview will take approximately 45-60 minutes. Is this still a good time to talk?

[If YES, continue with the interview; if NO, arrange another time to conduct the interview and thank them for their time]

I am going to ask you a series of questions about your eating habits. There are no right or wrong answers, and I hope you will share your opinions openly and honestly. The interview will be recorded so that we can keep an accurate record of your answers. Your responses will be shared with non-UAS researchers to understand eating habits but they will not have any other data collected by the UAS researchers. To protect your confidentiality, no names or other information that would identify you personally will be used in any reports based on today's discussion. Therefore, we ask that you do not mention your name or anyone else's name while I am recording.

Do you have any questions before we start?

[Answer any questions]

Section 1: General eating habits, food preferences, physical and psychological capability around food choices, and influencing factors.

First, I am going to ask you some questions about your eating habits and food preferences.

1) What types of foods do you typically eat throughout the day?
    - Why do you like to eat these types of foods?
    - How do you feel about the foods that you eat?
2) How hard or easy is it for you to eat the types of foods you would like to eat?
    - When you think of your ideal way of eating, what does that look like for you?
    - What makes it easier for you to eat the foods you would like to eat?
    - What makes it harder for you to eat the foods you would like to eat?
    - Are there any resources that you use to get the foods you want? For example, community gardens, government food programs, food pantries, or getting food from families and friends?
3) How do you describe your eating patterns? Do you use any labels to describe how you eat?
    - How do feel about describing your eating patterns?
    - What do the labels you use mean to you?

Section 2: Current meat consumption

1) Now, I'm going to ask you a bit about eating meat. Can you start by describing what kinds of meat you usually eat?

2) I'd also like to understand how much meat you eat. Some people eat meat multiple times per day, and others might eat it once or twice a week or year. Some people may never eat certain types of meat.
   - About how often you eat do meat? You can tell me the number of times per day, or per week, or per month.
   - About how often do you eat red meats like beef, lamb and pork? Again, you can tell me the number of times per day, or per week, or per month.
   - About how often do you eat red meats that are highly processed? This would include things like pork or beef sausages, bacon, or ham slices? Again, you can tell me the number of times per day, or per week, or per month.

3) How do you feel about the amount of meat you eat? Are you generally happy about the amount of meat you eat?
   - If you could change the amount or type of meat you eat, what kinds of changes would you make?
   - How do you feel about the amount of red meats like beef, lamb and pork, that you eat?
   - What has the biggest influence on the amount of meat you eat?
   - What are the main reasons why you eat meat?
   - How do the people around you impact the amount of meat you eat?
   - On a scale from 0-5 with 0 being not healthy at all and 5 being very healthy, how healthy do you think meat is?
   - On a scale from 0-5, with 0 being not healthy at all and 5 being very healthy, how healthy do you think red meat like beef, lamb and pork are?

Section 3: Meat reduction

1) Some people are reducing the amounts of meat they eat. For example, some people may make some meals without meat or use meat substitutes, or have days where they don't eat meat, like Meatless Monday. Some people may reduce the portion sizes of meat they eat. Have you ever considered reducing the amount of meat you eat?
   - [*If YES*]:
     - Tell me about what made you consider eating less meat.
     - How do you feel about eating less meat?
     - How has the amount of meat you eat changed since you considered reducing the amount of meat you eat? What influenced this change?
     - What made it harder to eat less meat? Why?
     - What made it easier to eat less meat? Why?

        - Who are the people in your life that make it harder to eat less meat? Why?
        - Who are the people in your life that make it easier to eat less meat? Why?
    - [*If NO*]:
        - How do you feel about reducing your meat intake?
        - How likely are you to consider reducing your meat intake in the future?
        - What would make it easier for you to eat less meat? Why?
        - What would make it harder for you to eat less meat? Why?
        - Are there people in your life who would make it easier or harder to eat less meat? Why?

2) Now, I want to ask specifically about reducing the amount of **red meat** you eat. Red meats include things like beef, lamb and pork. Have you ever considered reducing the amount of red meat you eat?
    - [*If YES*]:
        - Tell me about what prompted you to consider eating less red meat.
        - How do you feel about eating less red meat?
        - What made it harder to eat less red meat? Why?
        - What made it easier to eat less red meat? Why?
        - Are there people in your life who would make it easier or harder to eat less red meat? Why?
        - How has the amount of red meat you eat changed since you started considering reducing the amount of red meat you eat? What influenced this change?
    - [*If NO*]:
        - How do you feel about reducing your red meat intake?
        - How likely are you to consider reducing your meat intake in the future?
        - What would make it easier for you to eat less red meat? Why?
        - What would make it harder for you to eat less red meat? Why?
        - Are there people in your life who would make it easier or harder to eat less red meat? Why?

4) How healthy do you think it is to eat less meat? I'd like you answer on a scale from 1 to 5, where 1 is that you think it's not healthy at all to eat less meat, and 5 is that you think it is very healthy to eat less meat.
    - How do you feel about the healthiness of eating less meat?
    - What concerns do you have about your health when considering changing the amount of meat you eat?
    - How important do you think eating meat is for your overall health?
    - How important do you think eating meat is for protein intake?
    - How important do you think eating meat is for your overall energy?
    - How important do you think eating meat is for staying full?

5) If you were going to reduce the amount of meat you eat, do you think you would be able to do it? Why or why not?
   - Which kinds of meat would you reduce? Why?
   - Do you have the information you'd need on how to select or prepare meals that have less meat?
   - How do you think money impacts your ability to eat less meat?
   - How would things like taste or convenience impact your ability to eat less meat?
   - How do you think the people around you would react if you were trying to eat less meat?

6) In the next section we are going to talk about different strategies that might motivate some people to reduce the amount of meat they eat. I am going to describe 4 different types of strategies. Then I will ask how you feel about each one.
   - First: In some places, there are now labels on food packaging that indicate how environmentally sustainable a food is. The purpose of these labels is to tell the consumer if the food is likely to damage the environment. Some foods damage the environment more than others. If you saw a food item, like a hamburger, that was labeled as less sustainable or not sustainable, how would that impact your likelihood to buy and eat that food?
   - Now, Imagine a café where plant-based options are cheaper than meat options. For example, if you were eating out and a vegetarian burger was a couple dollars less than a meat burger. How would lower prices for plant-based options impact the foods you order and eat ?
   - A third strategy is that some programs give out boxes filled with grocery ingredients that are environmentally sustainable, so that customers can make sustainable, healthy meals and snacks at home. If you were offered a box of free sustainable groceries that had mostly plant-based foods with little to no meat in it, how would this impact what you eat?
   - Last, imagine you get a $50 coupon every week to buy sustainable grocery food items that include plant-based proteins like beans, tofu or meat substitutes, grains, fruits and vegetables. How would this impact what you eat?
   - Which of these ideas that I described sounds most appealing to you?
     Why would this strategy be effective for you?
   - Can you think of any other strategies or programs that would help you or encourage you to reduce the amount of meat you eat?

Section 4: Sustainable eating

1) How important do you think it is to think about how the foods you eat impact the environment?
   - When you think about the foods that you typically eat, how environmentally sustainable do you think the way you eat is?
   - What foods do you think are less environmentally sustainable, meaning they are more harmful to the environment?
   - What foods do you think are more environmentally sustainable, meaning they are better for the environment?

2) Have you ever heard of sustainable eating, or ways to make food choices that are better for the environment?
   - [*If YES*]:
     - Can you please describe what sustainable eating means to you?
     - Where did you hear about sustainable eating?
     - How do you feel about sustainable eating?
     - How interested are you in learning more about eating in a way that has no negative impacts on the environment?
     - How do you feel about thinking about how foods impact the environment?
   - [*If NO*]:
     - How interested are you in learning more about eating in a way that has no negative impacts on the environment?
     - How do you feel eating in a way that is sustainable?

3) How sustainable do you think it is to eat red meat? I'd like you to answer on a scale from 1 to 5, where 1 means eating red meat is not sustainable at all, and 5 means eating red meat is very sustainable.
   - How sustainable do you think meat is?
   - What types of meats do you think are less sustainable?
   - What types of meats do you think are more sustainable?
   - How sustainable do you think red meat is?

<u>Opportunity to add</u>: Thank you for your participation in today's survey. Do you have any thoughts on anything we talked about today, including eating patterns, sustainable food choices, or reducing meat intake that you would like to add?

<u>Concluding remarks</u>: Thank you so much for your time. We will distribute your coupon to your address or email address. What address or email address should we send it to?

[Confirm/record email address]
Thank you. Do you have any other questions at this time?

[Answer questions]

Have a great [*morning/afternoon*].

References:

United States National Institute of Health. (2008). *Usual Dietary Intakes: NHANES Food Frequency Questionnaire (FFQ)*.
