# Supplementary material for: Barriers and enablers to reduced meat intake and perceptions of sustainable diets, among Los Angeles County adults with low incomes: a qualitative interview study
Source: Front Public Health. 2026 Mar 26;14:1741901. doi: 10.3389/fpubh.2026.1741901 (PMC13064549; doi:10.3389/fpubh.2026.1741901)
Supplement: Supplementary file 2 [file Supplementary_file_2.pdf]

Observaciones preliminares:

Gracias por dedicar su tiempo a participar en esta entrevista. Me llamo *[nombre del investigador*] y hoy realizaré esta entrevista. Este estudio forma parte de un proyecto que pretende comprender los hábitos alimentarios.

Información logística:

Esta entrevista durará aproximadamente entre 45 y 60 minutos. ¿Sigue siendo un buen momento para hablar?

[Si la respuesta es SÍ, continúe con la entrevista; si la respuesta es NO, acuerde otro momento para realizar la entrevista y agradézcale su tiempo]

Voy a hacerle una serie de preguntas sobre sus hábitos alimentarios. No hay respuestas correctas o incorrectas, y espero que comparta sus opiniones abierta y honestamente. La entrevista se grabará para que podamos mantener un registro preciso de sus respuestas. Sus respuestas se compartirán con investigadores ajenos a la UAS para conocer los hábitos alimentarios, pero no dispondrán de ningún otro dato recogido por los investigadores de la UAS. Para proteger su confidencialidad, no se utilizarán nombres ni otros datos que puedan identificarle personalmente en ningún informe basado en la conversación de hoy. Por lo tanto, le rogamos que no mencione su nombre ni el de nadie mientras esté grabando.

¿Tiene alguna pregunta antes de empezar?

[Responda a cualquier pregunta]

Sección 1: Hábitos alimentarios generales, preferencias alimentarias, capacidad física y psicológica en torno a la elección de alimentos y factores que influyen.

En primer lugar, voy a hacerle algunas preguntas sobre sus hábitos alimentarios y sus preferencias alimentarias.

1) ¿Qué tipo de alimentos suele comer a lo largo del día?
    - ¿Por qué le gusta comer este tipo de alimentos?
    - ¿Qué opina de los alimentos que come?
2) ¿Le resulta difícil o fácil comer el tipo de alimentos que le gustaría?
    - Cuando piensa en su forma ideal de alimentarse, ¿qué aspecto tiene para usted?
    - ¿Qué le facilita comer los alimentos que le gustaría comer?
    - ¿Qué le hace que sea más difícil comer los alimentos que le gustaría comer?
    - ¿Hay algún recurso que utilice para conseguir los alimentos que desea? Por ejemplo, ¿huertos comunitarios, programas gubernamentales de alimentación, despensas de alimentos u obtener comida de familiares y amigos?

3) ¿Cómo describiría sus patrones alimentarios? ¿Utiliza alguna etiqueta para describir cómo come?
    - ¿Cómo se sientes al describir sus patrones alimentarios?
    - ¿Qué significan para usted las etiquetas que utiliza?

Sección 2: Consumo actual de carne

1) Ahora, voy a preguntarte un poco sobre el consumo de carne. ¿Puede empezar describiendo qué tipos de carne suele consumir?

2) También me gustaría saber cuánta carne consume. Algunas personas consumen carne varias veces al día, mientras que otras lo hacen una o dos veces a la semana o al año. Algunas personas no consumen nunca determinados tipos de carne.
    - ¿Con qué frecuencia come carne? Puede decirme el número de veces que consume al día, a la semana o al mes.
    - ¿Con qué frecuencia consume carnes rojas como carne de res, cordero y cerdo? De nuevo, puede decirme el número de veces que consume al día, a la semana o al mes.
    - ¿Con qué frecuencia consume carnes rojas muy procesadas? ¿Esto incluye productos como salchichas de cerdo o carne de res, tocino o jamón? De nuevo, puede decirme el número de veces que consume al día, a la semana o al mes.

3) ¿Qué opina de la cantidad de carne que consume? ¿Está contento en general con la cantidad de carne que consume?
    - Si pudiera cambiar la cantidad o el tipo de carne que consume, ¿qué tipo de cambios haría?
    - ¿Qué opina de la cantidad de carnes rojas, como carne de res, cordero y cerdo, que consume?
    - ¿Qué es lo que más influye en la cantidad de carne que consume?
    - ¿Cuáles son las principales razones por las que consume carne?
    - ¿Cómo influye la gente que le rodea en la cantidad de carne que consume?
    - En una escala de 0 a 5, siendo 0 nada saludable y 5 muy saludable, ¿en qué medida cree que la carne es saludable?
    - En una escala del 0 al 5, siendo 0 nada saludable y 5 muy saludable, ¿qué tan saludable cree que es la carne roja como la carne de res, el cordero y el cerdo?

Sección 3: Reducción en el consumo de carne

1) Algunas personas están reduciendo las cantidades de carne que consumen. Por ejemplo, algunas personas pueden hacer algunas comidas sin carne o utilizar sustitutos de la carne, o tener días en los que no consumen carne, por ejemplo, "Lunes sin carne". Algunas personas pueden reducir el tamaño de las porciones de carne que consumen. ¿Ha pensado alguna vez en reducir la cantidad de carne que consume?
    - [*En caso AFIRMATIVO*]:

    - Dígame qué le hizo plantearse consumir menos carne.
    - ¿Qué le parece consumir menos carne?
    - ¿Cómo ha cambiado la cantidad de carne que consume desde que se planteó reducirla? ¿Qué ha influido en este cambio?
    - ¿Qué hizo más difícil consumir menos carne? ¿Por qué?
    - ¿Qué hizo más fácil consumir menos carne? ¿Por qué?
    - ¿Quiénes son las personas de su vida que hacen más difícil consumir menos carne? ¿Por qué?
    - ¿Quiénes son las personas de su vida que le facilitan consumir menos carne? ¿Por qué?
- [*En caso NEGATIVO*]:
    - ¿Qué le parece reducir el consumo de carne?
    - ¿Qué probabilidades hay de que se plantee reducir su consumo de carne en el futuro?
    - ¿Qué le facilitaría consumir menos carne? ¿Por qué?
    - ¿Qué le dificultaría consumir menos carne? ¿Por qué?
    - ¿Hay personas en su vida que le facilitarían o dificultarían consumir menos carne roja? ¿Por qué?

2) Ahora, quiero preguntarle específicamente sobre la reducción de la cantidad de **carne roja** que consume. Las carnes rojas incluyen la carne de res, el cordero y el cerdo. ¿Ha pensado alguna vez en reducir la cantidad de carne roja que consume?
    - [*En caso AFIRMATIVO*]:
        - Cuénteme qué le llevó a plantearse consumir menos carne roja.
        - ¿Qué le parece consumir menos carne roja?
        - ¿Qué hizo más difícil consumir menos carne roja? ¿Por qué?
        - ¿Qué le facilitó consumir menos carne roja? ¿Por qué?
        - ¿Hay personas en su vida que le facilitarían o dificultarían consumir menos carne roja? ¿Por qué?
        - ¿Cómo ha cambiado la cantidad de carne roja que consume desde que empezó a plantearse reducirla? ¿Qué ha influido en este cambio?
    - [*En caso NEGATIVO*]:
        - ¿Qué le parece reducir el consumo de carne roja?
        - ¿Qué probabilidades hay de que se plantee reducir su consumo de carne en el futuro?
        - ¿Qué le facilitaría consumir menos carne roja? ¿Por qué?
        - ¿Qué le dificultaría consumir menos carne roja? ¿Por qué?
        - ¿Hay personas en su vida que le facilitarían o dificultarían consumir menos carne roja? ¿Por qué?

4) ¿Le parece saludable consumir menos carne? Me gustaría que respondiera en una escala del 1 al 5, donde 1 es que cree que no es nada saludable consumir menos carne, y 5 que cree que es muy saludable consumir menos carne.

- ¿Qué opina de lo saludable que es consumir menos carne?
- ¿Qué preocupaciones tiene por su salud a la hora de plantearse cambiar la cantidad de carne que consume?
- ¿Qué importancia cree que tiene consumir carne para su salud en general?
- ¿Qué importancia cree que tiene el consumo de carne para la ingesta de proteínas?
- ¿Qué importancia cree que tiene consumir carne para su energía general?
- ¿Qué importancia cree que tiene consumir carne para mantenerse saciado?

5) Si tuviera que reducir la cantidad de carne que consume, ¿cree que sería capaz de hacerlo? ¿Por qué sí o por qué no?
   - ¿Qué tipos de carne reduciría? ¿Por qué?
   - ¿Dispone de la información necesaria para seleccionar o preparar comidas con menos carne?
   - ¿Cómo cree que influye el dinero en su capacidad para consumir menos carne?
   - ¿Cómo influirían aspectos como el sabor o la comodidad en su capacidad para consumir menos carne?
   - ¿Cómo cree que reaccionaría la gente que le rodea si intentara consumir menos carne?

6) En la siguiente sección vamos a hablar de diferentes estrategias que podrían motivar a algunas personas a reducir la cantidad de carne que consumen. Voy a describir 4 tipos diferentes de estrategias. Luego, le preguntaré qué opina de cada uno.
   - En primer lugar, en algunos lugares ya hay etiquetas en los envases de los alimentos que indican que tan sostenibles son desde el punto de vista del medio ambiente. El objetivo de estas etiquetas es indicar al consumidor si el alimento puede dañar al medio ambiente. Algunos alimentos dañan el medio ambiente más que otros. Si viera un alimento, como una hamburguesa, etiquetado como menos sostenible o no sostenible, ¿cómo afectaría eso a su probabilidad de comprar y consumir ese alimento?
   - Ahora, imagine una cafetería donde las opciones basadas en plantas son más baratas que las de carne. Por ejemplo, si fuera a comer fuera y una hamburguesa vegetariana costara un par de dólares menos que una hamburguesa de carne. ¿Cómo repercutiría la bajada de precios de las opciones vegetales en los alimentos que pide y consume?
   - Una tercera estrategia consiste en que algunos programas repartan cajas llenas de ingredientes de comestibles sostenibles, desde el punto de vista medioambiental, para que los clientes puedan preparar en casa comidas y tentempiés sostenibles y saludables. Si le ofrecieran gratis una caja de alimentos sostenibles, en su mayoría de origen vegetal y con poca o nada de carne, ¿cómo afectaría esto a lo que consume?
   - Por último, imagine que recibe un cupón de 50 dólares cada semana para comprar alimentos sostenibles que incluyan proteínas vegetales como alubias, tofu o sustitutos de la carne, cereales, frutas y verduras. ¿Cómo afectaría esto su consumo?
   - ¿Cuál de las ideas que he descrito le parece más atractiva?
     ¿Por qué le resultaría efectiva esta estrategia?
   - ¿Se le ocurren otras estrategias o programas que le ayuden o animen a reducir la cantidad de carne que consume?

Sección 4: Alimentación sostenible

1) ¿Hasta qué punto crees que es importante reflexionar sobre el impacto medioambiental de los alimentos que consume?
   - Cuando piensa en los alimentos que consume habitualmente, ¿hasta qué punto cree que su forma de comer es sostenible para el medio ambiente?
   - ¿Qué alimentos cree que son menos sostenibles desde el punto de vista ecológico, es decir, que son más perjudiciales para el medio ambiente?
   - ¿Qué alimentos cree que son más sostenibles desde el punto de vista ecológico, es decir, que son mejores para el medio ambiente?

2) ¿Ha oído hablar alguna vez de la alimentación sostenible o de cómo elegir alimentos más respetuosos con el medio ambiente?
   - [*En caso AFIRMATIVO*]:
     - ¿Puede describir qué significa para usted una alimentación sostenible?
     - ¿Dónde escuchó hablar de la alimentación sostenible?
     - ¿Qué opina de la alimentación sostenible?
     - ¿Hasta qué punto le interesa aprender a comer de una forma que no tenga efectos negativos en el medio ambiente?
     - ¿Qué opina de la reflexión sobre el impacto de los alimentos en el medio ambiente?
   - [*En caso NEGATIVO*]:
     - ¿Hasta qué punto le interesa aprender a comer de una forma que no tenga efectos negativos en el medio ambiente?
     - ¿Cómo se siente comiendo de forma sostenible?

3) ¿Hasta qué punto cree que es sostenible comer carne roja? Me gustaría que respondiera en una escala del 1 al 5, donde 1 significa que comer carne roja no es sostenible en absoluto, y 5 significa que comer carne roja es muy sostenible.
   - ¿Hasta qué punto cree que la carne es sostenible?
   - ¿Qué tipos de carne cree que son menos sostenibles?
   - ¿Qué tipos de carne cree que son más sostenibles?
   - ¿Hasta qué punto cree que es sostenible la carne roja?

Oportunidad de añadir: Gracias por participar en la encuesta de hoy. ¿Tiene alguna opinión que añadir sobre algo de lo que hemos hablado hoy, como los patrones alimentarios, la elección de alimentos sostenibles o la reducción del consumo de carne?

Observaciones finales: Muchas gracias por su tiempo. Le enviaremos el cupón a su dirección postal o de correo electrónico. ¿A qué dirección o correo electrónico debemos enviarlo?

[Confirmar/registrar dirección de correo electrónico]
¡Gracias! ¿Tiene alguna otra pregunta en este momento?

Actualizado el 18/12/2023

[Responda a las preguntas]

Que tenga una buena [*mañana/tarde*].

Referencias:

United States National Institute of Health. (2008). *Usual Dietary Intakes: NHANES Food Frequency Questionnaire (FFQ)*.
